# Preliminary validation of the short physical performance battery in older adults with multiple sclerosis: secondary data analysis

**DOI:** 10.1186/s12877-015-0156-3

**Published:** 2015-12-03

**Authors:** Robert W. Motl, Yvonne C. Learmonth, Thomas R. Wójcicki, Jason Fanning, Elizabeth A. Hubbard, Dominique Kinnett-Hopkins, Sarah A. Roberts, Edward McAuley

**Affiliations:** Department of Kinesiology & Community Health, University of Illinois at Urbana-Champaign, 233 Freer Hall, Urbana, IL 61801 USA; Exercise Science Department, Bellarmine University, Louisville, Kentucky USA

**Keywords:** Multiple Sclerosis, Aging, Function, Validity

## Abstract

**Background:**

There are relatively few standard, objective measures for studying physical function among older adults with multiple sclerosis (MS), yet such measures are necessary considering the shift in prevalence and associated consequences of both MS and older age on physical function. We undertook a preliminary examination of the construct validity of Short Physical Performance Battery (SPPB) scores in older adults with MS based on an expected differential pattern of associations with measures of lower and upper extremity function.

**Methods:**

The sample included 48 persons with MS aged 50 years and older who were enrolled in a pilot, randomized controlled trial of exercise training. Participants completed the SPPB and other objective and self-report measures of lower and upper extremity function as part of baseline testing.

**Results:**

SPPB scores demonstrated strong associations with measures of lower extremity function (|*r*_s_| = .66–.79), and weak associations with measures of upper extremity function (|*r*_s_| = .03–.33).

**Conclusions:**

We provide preliminary evidence that supports the validity of scores from the SPPB as a measure of lower extremity function for inclusion in clinical research and practice involving older adults with MS.

## Background

Of the estimated 400,000 adults living with multiple sclerosis(MS) in the United States, 32 % are between the ages of 55–64 years and 14 % are 65 years of age or older [1]. There is additional evidence of a shift in the peak prevalence of MS among older age groups of adults (i.e., women 45–59 years of age and men 55–69 years of age) [2]. Importantly, this cohort undergoes normal age-related changes in function (e.g., ambulatory and balance dysfunction and leg muscle weakness) as well as those associated with MS and its progression [3]. Older adults with MS report limitations with both basic and instrumental activities of daily living [4], have accelerated rates of neurological disability progression [5], and express concerns about continuing loss of function and mobility with aging [6, 7]. Such observations underscore the importance of studying physical function among older adults with MS in both clinical and research settings.

**Fig. 1:**
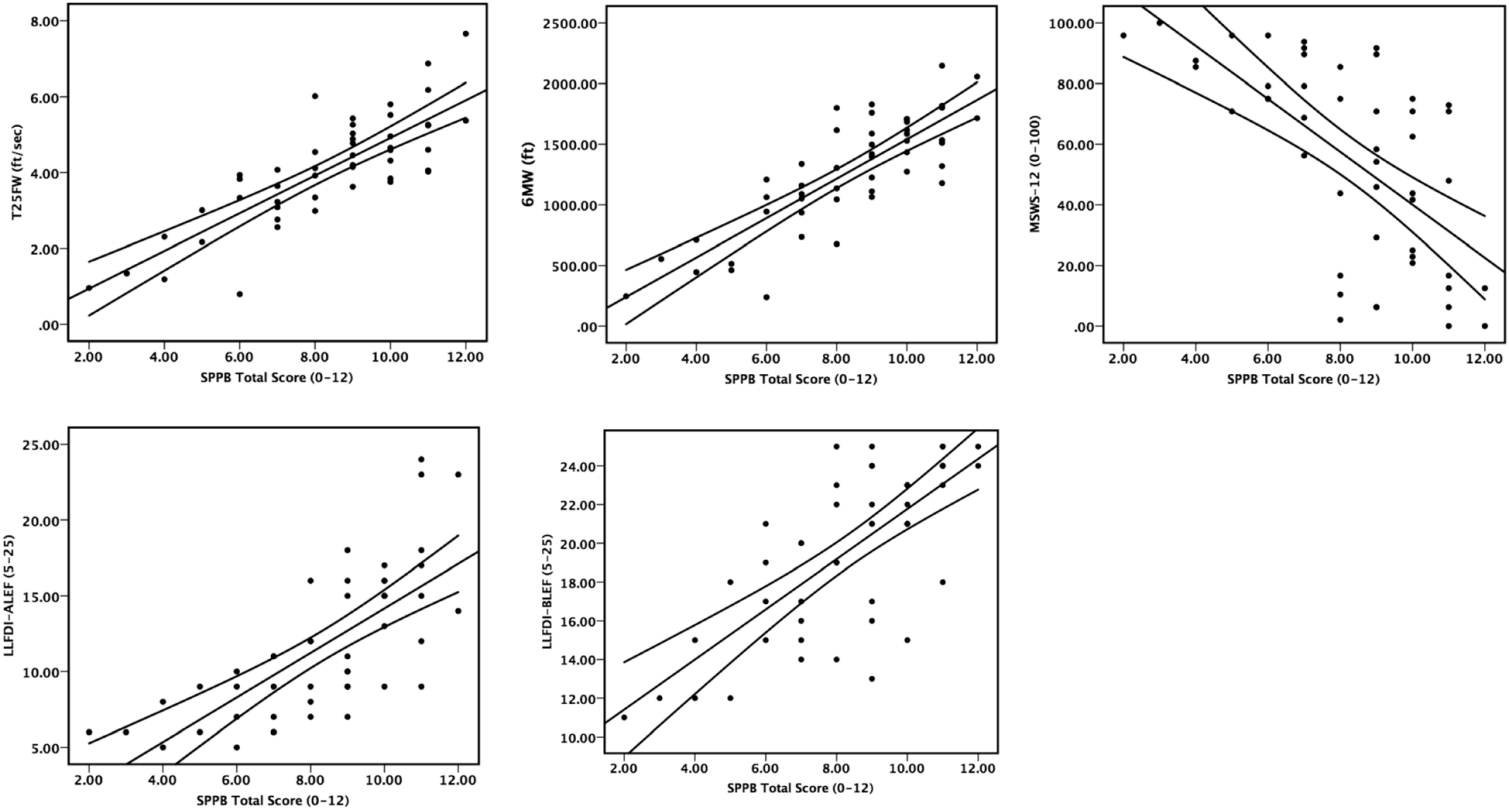
Scatter plots of the association between Short Physical Performance Battery(SPPB) scores and Timed 25--‐Foot Walking (T25FW), 6--‐Minute Walk (6 MW), Multiple Sclerosis Walking Scale--‐12 (MSWS--‐12), Late--‐Life Function and Disability Instrument, Advanced Lower Extremity Function Subscale (LLFDI--‐ALEF), and Late--‐ Life Function and Disability Instrument, Basic Lower Extremity Function Subscale (LLFDI--‐BLEF)scores

There are relatively few standard, objective measures for studying physical function among older adults with MS, and we believe that the Short Physical Performance Battery (SPPB) holds considerable promise as such an outcome assessment. The SPPB was developed as an objective measure for evaluating lower extremity function in older adults [8]. The SPPB includes assessments of balance, gait speed, and lower extremity strength, and those functions are compromised with the intersection of MS and aging. This battery can be administered easily, quickly (10 min), and uniformly in a variety of contexts by researchers and clinicians who complete a short course of training [9]. There is a standard system for scoring performance on the three assessments and those scores are aggregated into a summary SPPB score. The summary SPPB score has been associated with nursing home administration and mortality, as well as mobility and disability over a four-year period in community-dwelling older adults [8, 10]. Additionally, the SPPB has been included as a performance measure in hospitalized older adults [11] and clinical trials of exercise training among older adults [12], and has been recognized as the best performance-based measure of physical function for community-dwelling older adults [13].

The existing research on physical function in older adults with MS has included self-report measures [4, 6] and these seemingly have problems with validity, reproducibility, sensitivity to change, and applicability for cross-cultural and cross-national studies, as noted for normal aging populations [14]. To that end, we examined the construct validity (i.e., convergent and divergent validity) of SPPB scores in older adults with MS based on an expected pattern of correlations with objective and self-report measures of lower and upper extremity function. Convergent validity was based on expected strong correlations with measures of lower extremity function (i.e., 6-min walk [6 MW] [15], timed 25-foot walk [T25FW] [16], Multiple Sclerosis Walking Scale-12 [MSWS-12] scores [17], and the abbreviated Late-Life Function and Disability Instrument [LLFDI] basic [BLEF] and advanced lower extremity function [ALEF] subscale scores[18]). Divergent validity was based on expected weak correlations with measures of upper extremity function (i.e., arm curls, hand grip strength, and LLFDI upper extremity function [UEF] scores).

## Methods

### Participants

This paper involves a secondary analysis of baseline data from a recently completed pilot, randomized controlled trial (RCT) of a 6-month, DVD-delivered exercise intervention in older adults with MS [19, 20]. The registration number of the original trial was NCT01993095. The sample included 48 persons with MS aged 50 years and older [6] who were recruited from within a 50-mile radius of Champaign-Urbana, IL. We recruited participants from a mailing list of persons with MS in Illinois, a database of previous research volunteers, and a research advertisement posted on the website of the Greater Illinois chapter of the National Multiple Sclerosis Society (NMSS). The advertisements informed potential participants of a free, programmatic home-based exercise program that targets flexibility, strength, balance, and mobility in older adults with MS. The inclusion criteria for participation were: (a) definite diagnosis of MS that was confirmed in writing by the patient’s neurologist; (b) relapse free in the last 30 days; (c) ambulatory with or without assistance (i.e., walk independently or walk with a cane/rollator); (d) capable of engaging in systematic exercise without exacerbating any existing condition; (e) clearance for participation in physical activity by personal physician; (f) physical inactivity defined as two or fewer days of exercise per week over previous 6 months; (g) Expanded Disability Status Scale (EDSS) [21] score of 6.5 or less(i.e., constant bilateral assistance); and (h) and Modified Telephone Interview for Cognitive Status score above 21 [22]. All participants provided written informed consent before participating in the study.

### Measures

The complete list of outcomes from the parent RCT has been reported elsewhere [20], and we included selected measures for establishing convergent and divergent construct validation of SPPB scores. Importantly, the participants were permitted to use canes and walkers during the SPPB as well as all assessments involving lower extremity function (e.g., T25FW & 6 MW).

#### SPPB

The SPPB assesses lower extremity function based on a three-part assessment, including standing balance, gait speed, and lower extremity strength [8–10]. Standing balance was assessed by asking participants to maintain upright posture for up to 10 s per test while standing with feet in side-by-side, semi-tandem, and tandem positions. Those balance assessments occurred in a progressive order wherein participants passed one test in order to attempt the subsequent, more challenging test. Gait speed was assessed based on the time taken by a participant to walk a four-meter course at a normal pace. Lower extremity strength was assessed by a chair stand test in which participants were instructed to sit in and fully rise from a chair five times as quickly as possible, without using arms for support. Participants were first asked to attempt and complete a single sit-and-rise before beginning the entire chair stand test. Performance scores for each SPPB individual assessment and a summary score aggregating the individual assessments were calculated as per standard SPPB protocol. Each of the three performance assessments was assigned a categorical score ranging from 0(inability to complete a test) through 4(highest level of performance) using standardized scoring, and the summary ranging between 0 and 12 was calculated by summing the standing balance, gait speed, and lower extremity strength categorical scores [8–10]. Higher scores reflect better lower extremity function.

#### T25FW

The T25FW was administered as a measure of walking speed and has been identified as the best-characterized objective measure of ambulation in MS based on its psychometric properties [16]. Participants completed two trials, and walked as quickly and safely as possible, and the outcome was the mean of the two walks in feet per second.

#### 6 MW

The 6 MW was administered as a measure of walking endurance that is valid and reliable in persons with MS [15]. Participants completed the 6 MW by walking as fast and far as possible in a single corridor with two, 180° turns around cones separated by 75 feet. The outcome was total distance traveled in feet.

#### MSWS-12

The MSWS-12 is a 12-item patient-rated measure of the impact of MS on walking [17]. The 12 items on the MSWS-12 are rated on a scale ranging between 1 (*Not at all*) and 5 (*Extremely*). The total MSWS-12 score ranges between 0–100 and is computed by summing the individual item scores, subtracting the minimum possible score (12), dividing by the maximal score (48), and then multiplying the result by 100 [6]. Higher scores reflect greater perceived walking impairment.

#### Abbreviated LL-FDI

The functional component of the abbreviated LLFDI was included as a patient-reported measure of functional limitations that has been validated in persons with MS [18]. This outcome contains 15 items that are broken down into 3 subscales of UEF, BLEF, and ALEF. The 15-items were rated on a 5-point ordinal scale of 1 (*none*) to 5 (*cannot do*) and were reverse-scored. Scores were averaged to comprise composite UEF, BLEF, and ALEF measures. Scores for each five-item subscale range between 5 and 25, and higher scores reflect fewer functional limitations [18].

#### Arm curls

Upper extremity muscle strength and endurance was assessed with a 30-s arm curl test; this is a standard component of the Senior Fitness Test [23]. Participants sat near the side of a chair while holding a dumbbell (5 lb for females, and 8 lb for males), and completed as many arm curls as possible over a 30 s period. The outcome was number of repetitions performed in 30 s, and higher values indicate greater arm strength.

#### Hand grip strength

We further measured upper extremity function based on hand grip strength using a hand-held dynamometer (Jamar-Hydraulic Hand Dynamometer, Sammons Preston, Bollingbrook, IL, 60440, USA) [24]. Participants completed two assessments per hand in an alternating manner, keeping the arm being tested against the side or the body with the elbow flexed at 90°, and squeezing the hand-held device as hard as possible for 3 s. The outcome was force generated (i.e., pounds/inch^2^) per hand, and higher values indicate greater grip strength.

### Procedure

The procedure was approved by the Institutional Review Board at the University of Illinois, Urbana-Champaign, and all participants provided written informed consent before participating in study procedures. The data were collected during one session in a single clinical setting. Participants underwent a neurological examination by a Neurostatus Certified examiner for generating EDSS scores as a description of neurological disability status [21]. Participants further undertook the SPPB and other assessments. The order of tests was standardized and there was seated-rest between the administration of lower extremity outcomes (e.g., SPPB or T25FW) and upper extremity outcomes (e.g., arm strength) by completing ‘non-physically-active’ outcomes (e.g., questionnaires). Participants received $50 USD for completing the measures.

### Data analysis

All data were analyzed in SPSS Statistics, Version 22 (IBM Corporation, Armonk, NY). We provide descriptive characteristics of the measures as median and interquartile range (IQR). We conducted Spearman rho rank-order correlations (*r*_s_) between SPPB scores and scores from the other measures. We included only non-parametric correlations in the event of outliers, non-normality of distribution, and non-linear associations between variables [25]. Values for correlation coefficients of .1, .3, and .5 were interpreted as weak, moderate, and strong, respectively [26]. We further note that many of the variables departed from a normal distribution based on the Shapiro-Wilk test of normality or were ordered-categorical, and this further justified the approached for descriptive and correlational analyses.

## Results

### Sample characteristics

The demographic and clinical characteristics of the sample are presented in Table 1. The sample had a median age of nearly 60 years and was primarily composed of women. The sample had predominantly relapsing-remitting MS, a median disease duration of 20 years, and moderate MS disability based on the median EDSS score.

**Table 1:** Demographic and clinical characteristics of the 48 older adults with multiple sclerosis

Variable	Descriptive Statistic
Age (years)	59.5 (5.75)
Sex (n, % female)	36, 75 %
MS Type (n, %)	
Relapsing-Remitting MS	32, 66.7 %
Secondary Progressive MS	5, 10.4 %
Progressive MS	1, 2.1 %
Unknown/Missing	10, 20.8 %
EDSS score (0–10)	4.5 (2.5)
MS Duration (years)	20.0 (15.0)
Assistive Device Use (n, %)	
None	30, 62.5 %
Cane	10, 20.8 %
Walker/Rollator	8, 16.7 %

Note: Data are presented as median (interquartile range), unless indicated otherwise. MS = multiple sclerosis

*EDSS* Expanded Disability Status Scale

### Descriptive characteristics

Descriptive data on the SPPB and measures of lower and upper extremity function are presented in Table 2. Of note, the summary SPPB score ranged between 2 and 12, with a median of 9.0 (3.0). The summary SPPB scores were distributed for the sample as 2.1 % had a score of 2, 2.1 % had a score of 3, 4.2 % had a score of 4, 4.2 % had a score of 5, 8.3 % had a score of 6, 12.5 % had a score of 7, 12.5 % had a score of 8, 18.8 % had a score of 9, 16.7 % had a score of 10, 14.6 % had a score of 11, and 4.2 % had a score of 12. This indicates 0 % floor effects, and 4.2 % ceiling effects for summary SPPB scores in the current sample of older adults with MS.

**Table 2:** Descriptive characteristics of SPPB scores and measures of lower and upper extremity function in 48 older adults with multiple sclerosis

Category	Variable	*Median (IQR)*
Primary Outcome		
	SPPB (0–12)	9.0 (3.0)
	Balance (0–4)	4.0 (1.0)
	Gait Speed (0–4)	4.0 (1.0)
	Lower Extremity Strength (0–4)	1.0 (1.0)
Lower Extremity Function		
	T25FW (ft/sec)	4.1 (1.7)
	6 MW (ft)	1,312 (571)
	MSWS-12 (0–100)	65.6 (62.4)
	LLFDI-ALEF (0–25)	10.5 (7.0)
	LLFDI-BLEF (0–25)	21.0 (7.0)
Upper Extremity Function		
	Arm Curls (# reps)	12.0 (4.0)
	Grip Strength-R (lbs/in^2^)	59.0 (24.5)
	Grip Strength-L (lbs/in^2^)	56.5 (20.0)
	LLFDI-UEF (0–25)	18.5 (5.0)

*Note. IQR* interquartile range, *SPPB* Short Physical Performance Battery, *T25FW* Timed 25-Foot Walk, *6 MW* 6-min Walk, *MSWS-12* Multiple Sclerosis Walking Scale-12, *LLFDI-ALEF* Late-Life Function and Disability Instrument, Advanced Lower Extremity Function Subscale, *LLFDI-BLEF* Late-Life Function and Disability Instrument, Basic Lower Extremity Function Subscale, *LLFDI-UEF* Late-Life Function and Disability Instrument, Upper Extremity Function Subscale

### Convergent and divergent validity

The associations between SPPB scores and measures of lower and upper extremity function are presented in Table 3. Regarding convergent validity, SPPB scores demonstrated strong associations with other measures of lower extremity function(e.g., |*r*_p_| = .64–.82, |*r*_s_| = .66–.79). On the other hand, there were weak associations with measures of upper extremity function(e.g., |*r*_p_| = .11–.27, |*r*_s_| = .03–.33) suggesting divergent validity. Scatter plots of associations between SPPB scores and measures of lower extremity function are provided in Fig. 1.

**Table 3:** Correlations between SPPB scores and measures of lower and upper extremity function in 48 older adults with multiple sclerosis

Category	Variable	*r*_s_
Lower Extremity Function		
	T25FW	.77 (.63, .87)*
	6 MW	.79 (.65, .87)*
	MSWS-12	–.66 (−.46, −.79)*
	LLFDI-ALEF	.75 (.60, .85)*
	LLFDI-BLEF	.70 (.52, .82)*
Upper Extremity Function		
	Arm Curls	.33 (.05, .56)*
	Grip Strength-R	.03 (−.26, .31)
	Grip Strength-L	.03 (−.26, .31)
	LLFDI-UEF	.24(−.05, .49)

*Note:* Values are correlation coefficient (95 % confidence interval). *r*_*s*_ Spearman rho rank-order correlations. *Denotes statistical significance of correlation coefficient, *p* < 0.05. *SPPB* Short Physical Performance Battery, *T25FW* Timed 25-Foot Walk, *6 MW* 6-min Walk, *MSWS-12* Multiple Sclerosis Walking Scale-12, *LLFDI-ALEF* Late-Life Function and Disability Instrument, Advanced Lower Extremity Function Subscale, *LLFDI-BLEF* Late-Life Function and Disability Instrument, Basic Lower Extremity Function Subscale, *LLFDI-UEF* Late-Life Function and Disability Instrument, Upper Extremity Function Subscale

## Discussion

There are few standard, objective measures for studying physical function among older adults with MS, yet such measures are necessary considering the shift in prevalence [1, 2] and associated consequences of MS and older age combined on physical function [4, 6]. We undertook a preliminary examination of the construct validity of SPPB scores as an objective measure of lower extremity function in this segment of the MS population who are aging with a chronic, disabling neurological disease. Our preliminary results(i.e., pattern of correlations) provided evidence for the construct validity of SPPB scores as a measure of lower extremity function in older adults with MS. Of note, those older adults with MS who had better SPPB scores demonstrated faster T25FW performance and greater 6 MW distance, and reported less walking impairment on the MSWS-12 and fewer lower extremity functional limitations on the abbreviated LLFDI (i.e., convergent aspects of construct validity). By comparison, there were weak and/or non-significant associations between SPPB scores and objective and self-report measures of upper extremity function (i.e., divergent aspects of construct validity). Collectively, the current results provide preliminary evidence for the construct validity of SPPB scores as a measure of lower extremity function in older adults with MS, and support the possible inclusion of the SPPB in clinical practice and research involving this growing demographic of MS.

The median score of 9.0 in the current sample of adults with MS who had a median age of ~60 years approximated the mean score estimated for non-disabled, community-dwelling adults 71 years of age and older(estimated mean = 9.2) [10]. The distribution of SPPB scores further was consistent with that reported in previous research of non-disabled, community-dwelling older adults [27]. The median SPPB score in the current study was below the cut-off value of 10 indicating elevated risk for developing future disability [10, 27, 28]. Of note, the lower extremity strength component of the SPPB indicated larger decrements in physical function than did gait speed or balance. These data suggest that this sample of older adults with MS exhibited physical function comparable with that of a sample of older adults that was one decade older, and the largest decrement was in lower extremity strength. Such observations should be confirmed in direct, head-to-head comparison of older adults with MS and community-dwelling adults without MS or other neurological disease.

Of note, the data herein provide a secondary analysis for establishing preliminary evidence for the validity of SPPB scores in older adults with MS. The primary analysis of SPPB scores was undertaken in a RCT for capturing the effect of a home-based, exercise training intervention targeting strength and balance that was delivered through DVD on physical function [19]. The results of the RCT provide evidence for a small, clinically meaningful change in the SPBB for exercise training compared with control. Such data may support the responsiveness of the SPPB for capturing changes in physical function seen with an exercise training intervention.

There are many possible applications of the SPPB as a standardized, objective measure for understanding physical function in older adults with MS. One application involves prospective comparison with other samples of older adults for understanding the effect of aging in the context of MS on physical function. Another application involves the prediction of disability and disease progression as well as participatory outcomes in older adults with MS. We envision considerable application of the SPPB in clinical trials examining the effects of disease modifying treatments and rehabilitation therapy in older adults with MS. SPPB scores might even predict intervention responders (e.g., those who have smaller or larger likelihood of benefit), and our previous RCT [19] was not designed nor powered for such an endeavor. There is further value in the application of the SPPB in clinical practice. The SPPB could be adopted for characterizing physical function as well as documenting changes over time. Researchers and clinicians might consider using a recent virtual SPPB for ongoing measurement of physical function in older adults with MS [29].

There are important limitations of the current study. The paper was based on a secondary analysis of data that were not explicitly collected for the purpose of validating the SPPB. This is a limitation, as we would have included other measures such as gait, posturography, falls and falls self-efficacy, and activities of daily living when validating SPPB scores. The sample was recruited for a RCT of exercise training, and might have unique characteristics that restrict the application of the validity evidence amongst the broader population of older adults with MS. The sample size was relatively small, and this might influence the precision of the correlations for validity judgments. The sample further had intact cognitive function, and many persons ambulated without assistive devices; these observations further limit broad application of the validity evidence for SPPB scores. Such concerns could be overcome based on subsequent research using a larger sample of older adults who are recruited for a focal effort on the construct validity of SPPB score inferences.

## Conclusion

We provide preliminary evidence for the validity of the SPPB as a measure of lower extremity functional performance for inclusion in clinical research and practice involving older adults with MS.

## Abbreviations

MS: Multiple Sclerosis
SPPB: Short Physical Performance Battery
6 MW: 6-minute walk
T25FW: Timed 25-foot walk
MSWS-12: Multiple Sclerosis Walking Scale-12
LLFDI: Abbreviated Late-Life Function and Disability Instrument
BLEFm: Basic Lower Extremity Function
ALEF: Advanced Lower Extremity Function
UEF: Upper Extremity Function
USD: United States Dollars
IQR: Inter-quartile Range
*r*_s_: Spearman Rho Rank-Order Correlations
